# Energy level: an important determinant of success in vascular surgery

**DOI:** 10.1590/1677-5449.180001

**Published:** 2019-04-08

**Authors:** Frank J. Veith, Paulo Eduardo Ocke Reis

**Affiliations:** 1 New York University & The Cleveland Clinic, New York, NY, USA.; 2 Universidade Federal Fluminense – UFF, Departamento de Cirurgia Geral e Especializada, Rio de Janeiro, RJ, Brasil.

 Success and leadership in any profession is determined by many factors. Intelligence, creativity, interpersonal and communication skills, judgment, commitment, persistence, motivation and ability to focus are all important assets. One factor rarely considered but probably as important as any of these is an individual’s intrinsic or cultivated energy level. 

 Why is this so important? A high energy level allows one to outwork the competition by using his or her other assets to a greater degree and with more vibrancy than others. Let’s look at the example of a superstar in vascular surgery, the late Michael DeBakey. Dr. DeBakey, who for many years was probably the best known surgeon in the world, was reputed to work more than 14 hours a day and required little sleep. He was an innovative and busy surgeon. He published extensively and spoke widely around the world. He was a most effective leader who created a uniquely famous and efficient surgical service, which trained many of the leading vascular and cardiac surgeons around the world. On top of all these achievements, Dr. DeBakey managed to be a leader in his University, in governmental committees and in his specialty organizations like the Society for Vascular Surgery. He also served as a medical and surgical advisor to multiple US Presidents and other world leaders. To fill all these roles effectively, to perform his highly complex and demanding job, to deal with the stresses involved in all these many events and arenas, the energy level required to stay focused and function effectively had to be enormous – and it was. 

 Success and leadership in any chosen field is similarly largely dependent on one’s energy level, which allows him or her to display other assets better. To be successful and become a leader in an occupation like vascular surgery, one must have many assets. These include mastery of knowledge and technical aspects within the field, the ability to deal well with other people and to organize and lead a group of colleagues, the ability to communicate well orally and in writing and having the drive to see one’s efforts carried though to a successful conclusion. Demonstration of these assets requires organization, hard work, persistence and focused thinking. 

 To use and display these assets most effectively is where the importance of a person’s high energy level becomes apparent. Without such energy, an individual may achieve excellence in one or another aspect of their profession or business. However, to be a successful leader, one must excel in all or almost all aspects of their work. This cannot happen without a high level of energy which enables one to function effectively in at least most key areas of their field simultaneously. 

 Admittedly having a high level of energy is a God-given asset which only a few may have. However, by focusing one’s attention in a specified area, it is possible to maximize the energy that can be brought to bear on that area. If an individual zeros in on key parts of their professional activity, they can achieve the high level of energy in those areas needed to achieve professional success. 

 So if a young person wants to become a successful leader in vascular surgery, they should ask themselves three questions. First, are they willing to make the sacrifices to achieve excellence in the many areas required? Second, do they have the talents or assets to do so? And third and most importantly, do they have the energy level to excel in all or most of these areas simultaneously? If the answer to these 3 questions is yes, with a little luck along the way, success and leadership in vascular surgery is quite attainable. 

## Invited Commentary

### Discussant Dr. Paulo Eduardo Ocke Reis:

 In this issue of the Journal Vascular Brasileiro, Frank J. Veith reports his lecture: “What it Takes to Succeed in Vascular Surgery?” during the Pan American Congress 2018 at Rio de Janeiro, Brazil. Dr. Frank J. Veith is professor of Vascular and Endovascular Surgery at New York University & The Cleveland Clinic. I had the great opportunity to accompany Dr. Veith`s medical work daily during a period between 2004 and 2005, at Montefiore Medical Center in New York, and all subsequent years at the VEITHsymposium, I read a lot of his research and clinical publications, saw him in other congresses, meetings, lectures etc. I have been able, since then, to realize and learn how his ability to work, care directly for patients with arterial and venous vascular diseases, and his activities in disseminating knowledge are examples or instances of improving his specialty at all times. Certainly I agree that success and leadership in any chosen field is similarly largely dependent on one’s energy level which allows him or her to display other assets better. These other assets include mastery of knowledge and technical aspects within the field, the ability to deal well with other people and to organize and lead a group of colleagues, having the ability to communicate well orally and in writing and having the drive to see one`s efforts carried through to a successful conclusion. All these require organization, hard work, persistence and focused thinking. You dear professor seem to have all these attributes. So, when you talk about a person with a high level of energy, the name I have in mind is Frank J. Veith. The example that you set for us increases every day. I also admire the way you support and help younger vascular surgeons to improve themselves. In addition, you have supported the continuing struggle to establish the independence of Vascular Surgery as a separate defined specialty. 1 ^,^ 2 And finally you have helped to disseminate and update knowledge and advances in our specialty so that all vascular surgeons and others can practice the best most up-to-date effective and justified care for their patients. 3 So, I thank you for the opportunity to learn from you and from your example. 3 I also thank you on behalf of other young and not so young vascular surgeons who can profit from what you have contributed to our specialty. 
